# Mapping Ebola in wild animals for better disease control

**DOI:** 10.7554/eLife.04565

**Published:** 2014-09-19

**Authors:** Sebastian Funk, Peter Piot

**Affiliations:** 1 **Sebastian Funk** is in the Centre for the Mathematical Modelling of Infectious Diseases, London School of Hygiene & Tropical Medicine, London, United Kingdomsebastian.funk@lshtm.ac.uk; 2 **Peter Piot** is in the London School of Hygiene and Tropical Medicine, London, United Kingdom

**Keywords:** Ebola, species distribution modelling, boosted regression trees, disease mapping, Ebolavirus, niche based modelling, human, viruses

## Abstract

Identifying the regions where wild animal populations could transmit the Ebola virus should help with efforts to prepare at-risk areas for future outbreaks.

**Related research article** Pigott DM, Golding N, Mylne A, Huang Z, Henry AJ, Weiss DJ, Brady OJ, Kraemer MU, Smith DL, Moyes CL, Bhatt S, Gething PW, Horby PW, Bogoch II, Brownstein JS, Mekaru SR, Tatem AJ, Khan K, Hay SI. Mapping the zoonotic niche of Ebola virus disease in Africa. *eLife* **3**:e04395. doi: 10.7554/eLife.04395

**Image** Large regions of West and Central Africa (red) have the right environmental conditions for Ebola infection in wild animals

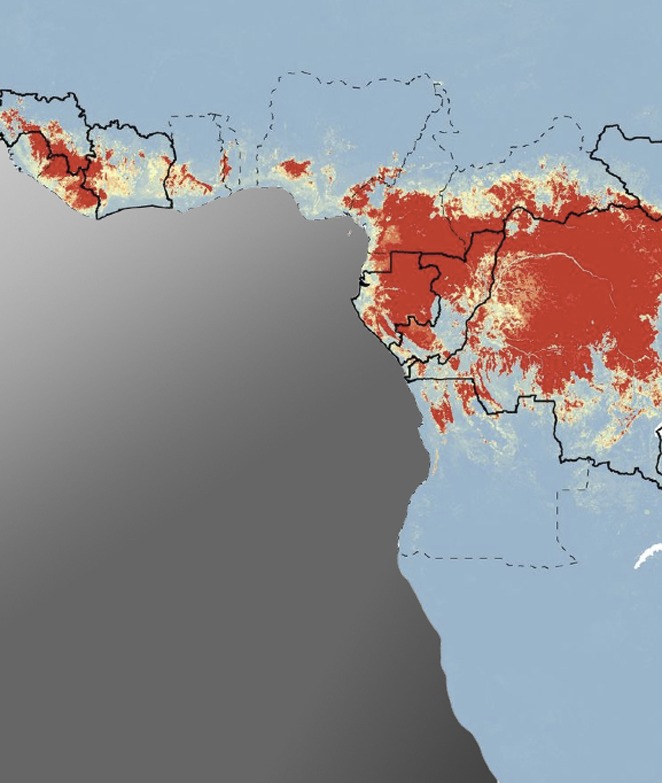

The scale of the current Ebola outbreak, the first ever to hit West Africa and the first to spread to urban areas, is unprecedented. Already more people have fallen ill and died than in all previously recorded outbreaks in Africa combined. The on-going outbreak now spans five West African countries (Guinea, Liberia, Nigeria, Sierra Leone and Senegal) and was recently declared a Public Health Emergency of International Concern by the World Health Organization. Health services are being overwhelmed, and the long-term social and economic consequences of the epidemic seem daunting.

The extent of the epidemic and the devastation it has caused has clearly, and rightly, dominated both the scientific discourse and media reporting. Yet, questions remain. How did the virus arrive in West Africa, a region that had no history of reported cases in humans? Where does the virus circulate between outbreaks? And where might future outbreaks strike? Now, in *eLife*, Simon Hay of the University of Oxford and co-workers in the UK, US, Canada and Sweden have mapped regions that are potentially at the most risk of a future Ebola outbreak (Pigott et al., 2014).

It has been known for years that several non-human species, notably primates and fruit bats, can be infected with the Ebola virus and that wild animals are the likely source of infection for outbreaks in humans. There have been 23 known human outbreaks of Ebola virus disease since its discovery in 1976 (Figure 1), but there have also been several large epidemics that have had devastating effects on wild primates (Walsh et al., 2005). For example, an outbreak of Ebola in a chimpanzee community in Côte d’Ivoire killed 25% of its members in the 1990s (Formenty et al., 1999). Because of the high mortality rate, primates are deemed an unlikely reservoir for the virus in the wild.

**Figure 1.**
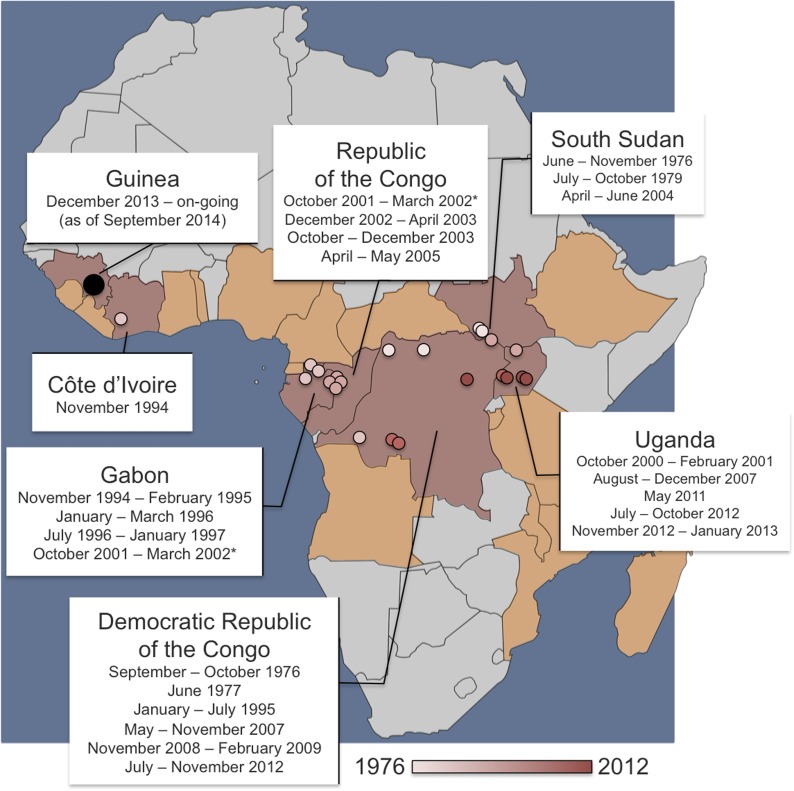
Dates and locations of outbreaks of Ebola virus disease in humans. Over the last four decades, there have been 23 outbreaks of Ebola virus disease in humans across Africa. The locations of the primary infections from past outbreaks are shown on the map as small circles (coloured pink or red according to the date that the outbreak started). The on-going outbreak in West Africa—which started in December 2013 in Guinea—is shown as a larger black circle. The 2001–2002 outbreak (marked with an asterisk) had primary infections (or ‘index cases’) in both the Republic of the Congo and Gabon—and the 1994 ‘outbreak’ in Côte d’Ivoire infected one researcher who had handled a dead chimpanzee and who later recovered. Pigott, Golding et al. have used the data of previous primary infections, reports of infections in wild animals and data about climate and vegetation to predict regions where Ebola might be transmissible from non-human animals. The at-risk area spans 22 countries: the seven with previous index cases (shaded in red) and a further 15 countries where, to date, index cases have not been recorded (shaded in orange).

Fruit bats, on the other hand, do not appear to fall ill when infected with Ebola. As such, bats are considered the most likely candidates for the reservoir species in which the virus lingers between outbreaks in humans. Antibodies against the Ebola virus have been found not only in three bat species in Central Africa (Leroy et al., 2005), but also in four bat species in West Africa (Hayman et al., 2012). Moreover, the start of at least one Ebola outbreak in humans has been linked to people consuming bat meat (Leroy et al., 2009). The Food and Agricultural Organization of the United Nations has now warned that rural communities in West Africa need to be made more aware of the risks of eating bats. Genetic analysis revealed that the current human outbreak probably originated in a child infected by a straw-coloured fruit bat (*Eidolon helvum;* Gire et al., 2014).

To predict where people might be at risk of Ebola outbreaks, it is important to know where the virus can circulate in wild animal populations, and this is exactly what Hay and co-workers—including David Pigott and Nick Golding of Oxford University as joint first authors—set out to do. After collecting information on all the reported Ebola cases in humans with a suspected origin in other species, and all reports of Ebola infections in non-human species from the last 40 years, they mapped the data across Africa. Hay and co-workers then matched the locations of these reports with a variety of environmental factors such as climate and vegetation, as well as with estimates for the distribution of fruit bats. The information obtained was used to plot a risk map of the potential for Ebola virus outbreaks in Africa.

The new map reveals that large swathes of Central and West Africa appear to have the right environmental conditions for Ebola infection to occur in non-human species.

Furthermore, when the analysis was repeated without using data about the on-going outbreak, an area deemed to be at risk was still within 5 kilometres of the village thought to the origin of the current outbreak.

This ‘at-risk area’ spans 22 countries and is inhabited by 22 million people. It should be stressed that this is not necessarily the size of the population at risk of getting infected with Ebola virus; rather, it represents the population that lives within the area estimated to be suitable for transmission in animals. Once an outbreak has started, transmission from human-to-human could easily spread the virus away from the source. This is an important issue given that the Democratic Republic of Congo—where most outbreaks so far have occurred—is currently home to more than 60 million people.

Given the large population found to be at risk of Ebola infection from other species, human outbreaks are rare. At the same time, when transmission does occur, a combination of factors are clearly able to cause an outbreak to grow and become as large as the one currently observed. These factors include: poorly prepared health care systems, a slow international response, and a lack of trust or even hostility towards control measures and care. Traditional beliefs and funeral practices that involve touching the body of the deceased can also contribute to the spread, as can the fact that there are now more people living in, and travelling between, the at-risk areas than ever before.

During the current emergency, caring for the sick and stopping the spread of the virus are clearly the most important and immediate tasks. Once the epidemic is over, however, it will be paramount to reassess the extent of the area at risk of outbreaks. Health care systems in these regions will then need to be strengthened and made capable of curbing any further outbreaks at the source. The area found to be at risk is home to some of the most under-resourced health care systems in the world—with the five countries afflicted by the current outbreak ranking among the world's worst both in terms of maternal mortality and human development (UN, 2014; WHO, 2014). Further investigation on the suspected and potential other animal reservoirs would refine the maps produced by Hay, Pigott, Golding and co-workers and thus help countries at risk to better prepare against Ebola outbreaks in the future.
